# The Ethical Tightrope of Physical and Environmental Restraint in ICU: A Balancing Act

**DOI:** 10.1111/nicc.70519

**Published:** 2026-05-14

**Authors:** Leona Bannon, Aisling Meehan, Natalie McEvoy

**Affiliations:** ^1^ School of Nursing Psychotherapy and Community Health Dublin City University Dublin Ireland; ^2^ School of Medicine University College Dublin Dublin Ireland; ^3^ Royal College of Surgeons Ireland Dublin Ireland

## Abstract

Physical and environmental restraints are synonymous with intensive care unit (ICU) practices in many countries around the world. The ICU environment is highly complex, and it is essential to maintain patient safety, but this can often be to the detriment of patients' autonomy. This article examines the ethical challenges that arise from the use of physical and environmental restraints in the intensive care unit (ICU). The article provides insights from both the patient and clinician perspectives using a real‐life‐informed fictional case and aims to shed light on the intricate ethical landscape surrounding these practices. It highlights the need to carefully balance patient autonomy, safety and clinical priorities when navigating these complex ethical dilemmas.

## Impact Statements

- What is known about the topic
  ○ Physical restraints affect 40%–50% of ICU patients globally, with chemical sedation compounding risks like delirium and PTSD; recent reviews show incomplete adoption of reduction strategies despite guidelines.
- What this paper adds
  ○ This paper employs a real‐life‐informed fictional case to dissect powerlessness across restraint types, offering referenced protocols, alternatives and ethical admission tools aligned with Irish, United Kingdom and wider European practice to support immediate clinical application*.*

## 1 Introduction

Physical restraint is defined as ‘any manually applied method that immobilises a person or reduces their ability to move any part of their body’ [1]. An integrative review conducted in 2020 indicated that 23%–75% of patients were restrained during their ICU stay [2]. While physical restraint practices are common in ICUs, questions have been raised regarding the potential for harm associated with physical restraint [3]. However, the persistence of physical restraint practices globally is shaped by several key factors, such as staffing levels, patient agitation and delirium, which warrant closer examination to mitigate associated harms [2]. Lack of adequate staffing is frequently cited as the most common rationale for nurses’ decisions to use physical restraints or chemical sedation, as it limits the capacity for alternative interventions such as one‐on‐one supervision or environmental modifications [4]. A recent systematic review and meta‐analysis showed an overall prevalence of physical restraint use at 41.6% with physical restraint being linked to delirium, sedation and mechanical ventilation [4, 5]. Similarly in acute wards in the US, research found that lower proportions of registered nurses per shift correlated with up to 18% higher restraint rates, as nurses are often better equipped to pursue non‐restraint alternatives than support staff. These findings underscore how staffing shortages compel time‐constrained decisions favouring restraints despite ethical concerns, highlighting the need for policy interventions to optimise nurse‐to‐patient ratios in critical care.

While physical restraint is often initiated to maintain a safe environment for the patient, it is also known to be associated with serious negative outcomes. Physical restraint use is associated with longer mechanical ventilation duration, development of delirium and even post‐traumatic stress disorder (PTSD) [6]. Physical restraint use is known to be higher in patients with delirium (77% vs. 50%, *p* < 0.05) but it is unclear where the association is derived [4].

The rationale for applying physical restraints may be to maintain lifesaving therapies, however, it does not always achieve its intended outcome. One study found that up to 44% of patients who performed self‐initiated device removal in the ICU had physical restraints in situ at the time [7]. Nurses are often the primary decision makers when it comes to applying or removing restraints [8]. This suggests that nurses play a central role in determining when restraints are used. One study explored the experience of physical restraint during mechanical ventilation among ICU patients and found that this leads to traumatic experiences which can impact patients and families post‐ICU discharge [9].

On the other hand, environmental restraint is defined as the ‘intentional restriction’ of ‘access to the environment, normal means of independent mobility and communication’ and ‘ability to exercise civil or religious liberties' [10]. While this situation is often associated with care of the older person, it can also be applicable to the ICU environment where patients are restricted by lifesaving therapies. Environmental restraint arises implicitly through ICU admission decisions, where ‘best interest’ determinations (as per relevant Irish, UK and European legislation) must weigh dehumanising elements against benefits, incorporating advance directives or lasting power of attorney to preserve liberty. Staff weigh capacity, least‐restrictive options and family input pre‐admission [11]. While the rationale for implementing restrictions may at times be clinically justified, such actions must be undertaken in strict accordance with legal and ethical standards that safeguard patient rights, and in most jurisdictions require the consent or authorisation of a legal representative [12, 13, 14].

This article will explore the ethical implications of the use of environmental restraint in ICU by providing a fictional patient scenario which has been informed by the lived experience of an anonymous former ICU patient. The former patient contributed to the development of the scenario but requests to be unnamed. This approach ensures that the ethical issues explored are grounded in authentic patient perspectives while maintaining confidentiality.

## 2 Ethical Discussion

### 2.1 Beneficence and Non‐Maleficence

Beneficence is the obligation to act in the best interest of the patient; meanwhile non‐maleficence is the obligation to do no harm [15]. While nurses aim to do no harm in their practice, it is estimated that as many as 1 in 10 patients experience an adverse event while receiving hospital care [16]. Restraint is often enacted to protect the patient from harm and is often implemented in the best interests of the patient and must be proportionate with the likelihood of severity of likely harm occurring [10]. However, there is a balancing act between maintaining safety and minimising the risk of harm. Nurses must navigate the delicate balance between protecting patients from harm through necessary environmental controls, while also ensuring that these interventions do not inadvertently cause psychological distress or diminish dignity for the patient.

Balancing harms and benefits of restraints in ICU requires prioritising non‐pharmacological delirium prevention (e.g., ABCDEF bundle elements: light sedation, delirium screening, early mobility), which reduces physical restraint exposure and invasive ventilation days more reliably than early pharmacological therapy, unsupported by recent trials and guidelines due to risks of oversedation [17, 18]. Minimising exposure to physical restraint is important as it can lead to PTSD among ICU survivors as well as prolonged duration of mechanical ventilation [3]. On the other hand, physical restraint is associated with a reduction in falls and can prevent accidental removal of medical devices [19]. This is a clear example of this ethical balancing act in terms of the use of physical restraint in ICU. Minimising the use of restraint is crucial, as they have been linked to adverse outcomes. However, their use can also serve a protective function.

The use of physical restraints not only affects patients but also presents a significant emotional and ethical impact on nursing staff. Nurses often experience a range of negative emotions, including guilt and moral conflict when using physical restraints, particularly due to concerns about infringing on patients' human rights [20]. This emotional toll is compounded by the responsibility nurses carry in maintaining a safe environment, not only for patients but also for families, colleagues and themselves. Navigating these competing demands places nurses at the heart of the ethical tension surrounding restraint use in the ICU [15].

Staffing levels also play a critical, yet often under‐acknowledged, role in shaping restraint decisions in ICU practice. As outlined in the introduction, inadequate nurse‐to‐patient ratios are consistently associated with higher restraint use, as reduced staffing limits the feasibility of labour‐intensive alternatives such as one‐to‐one supervision, early mobilisation or enhanced communication support. This relationship has been repeatedly highlighted in recent UK and European workforce reports, which warn that stretched staffing undermines the delivery of least‐restrictive care and increases reliance on restrictive measures that compromise autonomy. European policy analyses emphasise that safe staffing ratios are foundational to ethical, rights‐based care and essential for reducing avoidable restrictive practices [21]. Similarly, the UK Critical Care Nursing Alliance Workforce Optimisation Plan (2024–2027) [22] stresses that insufficient staffing directly constrains clinicians' ability to implement restraint‐minimisation strategies, while the updated Guidelines for the Provision of Intensive Care Services [23] reiterate that safe ratios are a prerequisite for humane, patient‐centred critical care. Within this ethical landscape, staffing pressures become more than an operational issue; they directly influence the balance between beneficence and autonomy by limiting clinicians' capacity to choose less restrictive options.

### 2.2 Justice and Autonomy

Autonomy is defined as ‘the capacity to make decisions and take actions that are in keeping with one's values and beliefs’ [13]. One of the ways that autonomy is upheld in clinical practice is the use of informed consent. In the United Kingdom (UK), each National Health Service (NHS) trust has their own consent policy which aligns with national guidelines and laws [13]^.^ In Ireland, the HSE National Consent Policy provides a framework for healthcare workers to ensure patients autonomy and rights to make informed decisions about their care is respected [14]. Physical and environmental restraints can limit a patient's freedom of movement, restrict their ability to communicate and participate in their care and infringe upon their autonomy and dignity [24]. There is consensus between nurses, patients and their family members that the application of physical restraints can lead to violation of patients' autonomy and dignity [6, 21]. Interestingly, patients and family members also understood that physical restraint was used for their protection but found it was overused and had the potential to cause physical injuries, safety issues and psychological distress [6, 24].

From a justice perspective, the use of physical restraints raises concerns about fairness, equality and the distribution of resources in health care. Patients who are restrained may experience a loss of control over their bodies and treatment decisions, leading to feelings of vulnerability and disempowerment. This loss of autonomy can impact a patient's sense of justice, as they may feel that their rights and preferences are not being respected or they are not being considered in the decision‐making process [13, 14]. The use of physical restraints can also disproportionately impact certain patient groups, for example, the elderly [25]. Ensuring equitable treatment is crucial to protect against abuse and ensure patients' rights are protected. Legally, in England and Wales, physical restraint is justifiable prior to formal authorisation, as long as the restraint use is to maintain life‐sustaining treatment intervention or prevent harm [13]. In Ireland, a combination of legal, regulatory and ethical guidelines governs the use of physical restraints [13, 26]. As Zhou and colleagues acknowledge, it is a tender balance between the rights of the patient to informed consent and the rights of healthcare providers to deliver essential treatment [24].

## 3 Clinical Scenario in Context

In the busy, high stakes environment of the ICU, patients often experience a stark and distressing loss of control. This loss of autonomy is not only a physical reality for the patient but also a conflict between the medical duty of the clinician and patient rights, impacting emotional well‐being, personal identity and the fundamental principles of patient‐centred care. Although ICU care prioritises survival, the human cost of eliminating agency must not be overlooked.

This was the reality for Malcolm, a young man in his 30s admitted to the ICU after a serious car accident. Surrounded by machines and monitored around the clock, Malcolm lay in bed unable to communicate, move freely or make decisions about his care. The mechanical beeping of monitors, the presence of unfamiliar medical staff and the invasive procedures imposed on him contributed to a sense of vulnerability and isolation. As sedation and mechanical ventilation removed his ability to speak, even basic needs or discomforts went unexpressed. For patients like Malcolm who remain cognitively aware, this voiceless state can be terrifying. It leaves a feeling of complete erasure of self‐determination and personal agency. Ethically and legally, there are alternative options for Malcolm to explore, such as clinician‐supported yes–no signals, communication boards, or other augmentative and alternative communication (AAC) methods, which are recognised in critical care guidance as essential tools for enabling consent and refusal in non‐speaking but cognitively intact ICU patients [27]. In addition, recognising that he retains decision‐making capacity even when silent, and aligning with Irish and British consent guidance, valid consent should be sought and documented in a way that respects his autonomy and understanding [13, 14].

ICU survivors often reflect on their time in critical care not merely as a medical ordeal, but as a deeply dehumanising experience [28]. Patients become passive recipients of interventions, being intubated, repositioned or restrained, often without consent or awareness [29]. The physical ICU environment can itself operate as a form of environmental restraint, where harsh lighting, constant noise and intrusive monitoring and life‐support equipment eclipse patients' dignity and effectively reduce them to physiological data points. This depersonalised milieu has been associated with negative outcomes [30]. Humanisation initiatives, such as designated quiet hours and the use of personalised communication or memory boards, have been shown to partially counter these effects by restoring a sense of personhood, control and relational connection within the critical care setting [31].

For Malcolm, and countless others, the path to regaining control must begin while still in critical care. This includes finding ways to reintroduce autonomy through even small acts like involving patients in care decisions when possible, using alternative communication tools, or simply explaining procedures clearly and empathetically [27]. The healthcare team must continually reassess their approach, seeking a delicate balance between what is medically necessary and what is ethically respectful. Communication, empathy and shared decision‐making become essential tools in restoring dignity and agency. By keeping the emotional and ethical needs and rights of an ICU patient at the forefront of their care, ICU clinicians can create a less dehumanising experience for their patients [32, 33].

Post‐Intensive Care Syndrome impacts 25%–50% of survivors with higher rates of PTSD, depression and anxiety [34]. Humanisation initiatives, like quiet hours and personal whiteboards, effectively mitigate these effects alongside nurse education and protocol‐driven restraint minimisation [6, 35, 36]. Evidence‐based protocols guide restraint decisions via standardised tools, with mandatory safety checks for skin integrity and circulation every 2 h and regular multidisciplinary review. Guide‐based interventions cut PR use by 40%–70% without increasing complications, reinforcing that safer, more person‐centred care is achievable when practice is aligned with robust evidence and continuous audit [37].

From an ethical standpoint, the dilemma centres on the tension between beneficence and autonomy. Medical interventions in the ICU are undeniably focussed on preserving life and improving health outcomes. However, these life‐saving measures often infringe on the patient's right to self‐determination. Sedation (chemical restraint) and physical restraints may both be medically necessary in acute scenarios to prevent device removal, but they similarly remove the patient's ability to consent, refuse or participate in their care. Sedation often correlates with longer mechanical ventilation durations (e.g., due to oversedation delaying weaning) and higher delirium incidence, while physical restraints, when used without adequate sedation, may independently elevate PTSD odds. However, combined use exacerbates both, underscoring the need for multimodal minimisation strategies like the ICU Liberation Bundle [6, 18, 38].

Ethical ICU care must therefore extend beyond clinical effectiveness to include the ways patients are ‘held’. Not only physically but also morally and emotionally within the system [39]. When patients are unable to speak or move, they are dependent on the ethical sensitivity of their caregivers. Trust becomes vital, but it is not mutual. It must be assumed and honoured by clinicians who may never know the person behind the patient. This creates a heightened moral responsibility for healthcare professionals to see the patient not only just as a body in crisis but also as a whole person [40]^.^

The lived experience of powerlessness in the ICU often leaves survivors with long‐lasting emotional scars, including PTSD, anxiety and depression persisting well beyond discharge. These patients frequently recall not just their illness but the profound helplessness and indignity from loss of autonomy, reinforcing the need to evaluate how ICU environments affect both immediate survival and long‐term psychological well‐being. Preserving life is insufficient if the process degrades the person's dignity and humanity in enduring ways [6, 27, 34].

Ultimately, the ICU presents a complex ethical landscape where the principle of beneficence can unintentionally undermine autonomy. Yet the two need not be in opposition. With conscious effort, ICU teams can uphold both, ensuring that patients are not only treated but also respected. Malcolm's experience is a compelling reminder that behind every monitor and machine lies a human being. One whose rights, identity and dignity deserve just as much attention as their medical needs. Patients like Malcolm exemplify how autonomy, dignity and agency are essential components of healing, not luxuries to be suspended during crisis. As clinicians, acknowledging and addressing this loss of control is vital in providing care that is not only effective but also humane.

## 4 Conclusion

The ICU, by its very nature, imposes significant restrictions on patient autonomy through both physical and environmental restrictions. While such measures are often clinically justified and aimed at preserving life, they can come at the cost of a patient's dignity, identity and psychological well‐being. The case of Malcolm illustrates how a life‐saving setting can also become a space of profound vulnerability and voicelessness for a patient. Ethical ICU care must therefore engage with more than physiological survival, and it must actively seek to minimise harm to the patients receiving care. Recognising patients as human beings with values and rights is not an added luxury but a moral imperative. By fostering communication, involving patients wherever possible, and being conscious of their emotional and ethical needs, nurses and clinicians can navigate the tension between beneficence and autonomy with compassion and integrity.

## Funding

The authors have nothing to report.

## Ethics Statement

The authors have nothing to report.

## Consent

The authors have nothing to report.

## Conflicts of Interest

The authors declare no conflicts of interest.

## Data Availability

Data sharing not applicable to this article as no datasets were generated or analysed during the current study.
